# ArcTiCA: Arctic tidal constituents atlas

**DOI:** 10.1038/s41597-024-03012-w

**Published:** 2024-02-03

**Authors:** M. G. Hart-Davis, S. L. Howard, R. D. Ray, O. B. Andersen, L. Padman, F. Nilsen, D. Dettmering

**Affiliations:** 1 Deutsches Geodätisches Forschungsinstitut, Technische Universität München, Munich, Germany; 2 Earth and Space Research, Seattle, WA USA; 3 Geodesy & Geophysics Lab., NASA Goddard Space Flight Center, Greenbelt, Maryland USA; 4 National Space Institute, Technical University of Denmark, Kongens, Lyngby Denmark; 5 Earth and Space Research, Corvallis, OR USA; 6 The University Centre in Svalbard, Longyearbyen, Norway

**Keywords:** Physical oceanography, Physical oceanography

## Abstract

Tides in the Arctic Ocean affect ocean circulation and mixing, and sea ice dynamics and thermodynamics. However, there is a limited network of available *in situ* tidal coefficient data for understanding tidal variability in the Arctic Ocean; e.g., the global TICON-3 database contains only 111 sites above 60°N and 21 above 70°N. At the same time, the presence of sea ice and latitude limits of satellite altimetry complicate altimetry-based retrievals of Arctic tidal coefficients. This leads to a reliance on ocean tide models whose accuracy depend on having sufficient *in situ* data for validation and assimilation. Here, we present a comprehensive new dataset of tidal constituents in the Arctic region, combining analyses of *in situ* measurements from tide gauges, ocean bottom pressure sensors and GNSS interferometric reflectometry. The new dataset contains 914 measurement sites above 60°N and 399 above 70°N, with each site being quality-assessed and expert guidance provided to help maximise the usage of the dataset. We also compare the dataset to recent tide models.

## Background & Summary

Ocean tides play a crucial role in large-scale ocean circulation through their role in mixing at the seabed and in the stratified interior^1^. In the Arctic Ocean, tides also influence sea ice distribution, cause the formation of leads within pack ice and play an important role in mixing and stirring deep Arctic Ocean waters, especially at the boundary between shelf and deep-basin water masses^2–4^. Around Greenland, tides contribute to mass loss from the ice sheet and the few remaining ice shelves^5^. The periodic rise and fall of ocean tides is the principal source of noise in sea-level estimations from satellite altimetry^6^, and the tidal redistribution of mass in the ocean is crucial for a variety of gravity modelling applications^7^. Tidal ranges in the Arctic Ocean are generally below one meter, but with much larger ranges in Baffin Bay, the White Sea, and the Davis Strait^8,9^.

Our current understanding of the spatial variability of Arctic Ocean tides comes primarily from models and, more recently, from satellite altimetry. However, modelling Arctic tides is complicated by substantial regions of sparse bathymetric data^10^, and complex interactions between tidal currents and sea ice (including landfast ice) that dampen propagating tidal energy to modify amplitudes and shift phases of tidal coefficients^11,12^. The seasonality of the Arctic sea ice^12^ including landfast ice^13^ has been shown to impact amplitudes and phase lag estimations for tidal constituents. Additional challenges arise because much of the tidal energy in the eastern Arctic is in the form of diurnal, topographically-trapped vorticity waves^14^, which are sensitive to small-scale topographic variability^15^ and background stratification and mean flows^16^. While assimilation of satellite altimetry can significantly improve tidal solutions^17^, these data have several limitations imposed by their orbit characteristics, footprint size (for radar altimeters) and contamination by sea ice and land.

In contrast to satellite altimetry, tide gauges provide fully resolved (unaliased) measurements, usually adjacent to the coast. Tide gauges have been used for centuries for both sea-level monitoring and ocean tide predictions^18,19^ and were essential resources for early global tide modelling efforts^20^. Even well into the altimetry era, tide gauges remain a crucial data source, particularly in the Arctic Ocean. For models purely constrained by satellite altimetry, tide gauges are a critical, independent data set for validation. Several state-of-the-art tide models also assimilate tidal analyses from *in situ* gauges, with clear benefits for model accuracy [^9,17,21–23^, for example], especially in regions with limited altimetry coverage. *In situ* tide records also provide insights into less energetic tidal coefficients which cannot be easily addressed with modern satellite altimetry data, e.g. higher harmonics in shallow water^24^, as well as tidal dynamics in estuarine systems^25^. Tide gauges can provide sea-level measurements all year round and, therefore, can be used to determine the fine-structure of the tidal spectrum, including the separation of constituents of nearby frequencies (e.g., S_2_ and T_2_).

However, for conventional altimetry missions, which have a footprint between 100 and 250 km^2^, this is not always possible due to gaps in the retrievals of sea level anomalies caused by the significant influence of sea ice. Tide gauges are usually limited to coastal regions, underscoring the utility of ocean bottom pressure (OBP) sensors to study ocean tides^26^ in other regions. These data measure shelf and open ocean, providing crucial insight into tidal dynamics, especially when coupled with the coastal measurements from tide gauges. In the Arctic Ocean, the placement and maintenance of both *in situ* measurement types are often complex, not to mention costly, based on the harsh conditions of sea ice and weather patterns. Satellite altimetry has resulted in significant improvements in the modelled prediction of ocean tides in the open ocean and in shelf and coastal regions ^17,26–28^, for example]. Improved altimetry processing and spatial and temporal coverage continue to drive advances in modelled tides. However, challenges remain in the Arctic regions^8^ due to limited altimetry missions in the Arctic Ocean and the sun-synchronous orbit of many of the missions that do orbit sufficiently far north. The geopolitical sensitivity relating to bathymetry information in the Arctic is another limitation, resulting in a lack of high-resolution bathymetry data for accurate numerical modelling of ocean tides^9^.

Solving all of the issues mentioned above, as well as those not mentioned or not yet identified, to fully understand ocean tides in the Arctic Ocean requires a concerted and collaborative effort to understand the complex tidal dynamics, as well as significant advances in computational resources and improved coverage of the appropriate observational measurements. Although these are challenging prospects, a first step towards these ambitious targets is to provide a harmonised and up-to-date *in situ* dataset that models can rely on for validation or assimilation purposes. The GESLA-3 tide gauge database^29^, used to determine tidal constituents (TICON-3^30^), contains only 21 stations above 70°N, with the distribution of gauges being mainly around the North American and Norwegian regions. Additional individual sources of constituents in the Arctic are available, but they provide inconsistent results from one another, and they are often difficult to retrieve and interpret. Furthermore, previous data not exploited for tidal estimations, notably from OBP sensors and GNSS-IR, are also publicly available.

This manuscript describes a common, documented source of tidal constituents from multiple sources and measurement types in the Arctic Ocean. The resultant dataset – the Arctic Tidal Constituents Atlas (ArcTiCA) – considerably increases the number of observations in the Arctic Ocean region compared with previous publicly available datasets. It is provided in two easy-to-use formats, comma-separated variable (CSV) and NetCDF, and can be subsetted based on user requirements. The ArcTiCA dataset and this manuscript complement the work on tidal currents from mooring observations in the Arctic Ocean by Baumann *et al*.^31^ but with a focus on tidal heights. The focus of this dataset is the Arctic Ocean, but data is provided down to 50°N to allow for ease of use of the dataset within Arctic tide model evaluations, whose boundaries often extend that far south^9,32,33^. ArcTiCa will be a valuable dataset for future scientific research into improved modelling of the tidal dynamics in the Arctic Ocean for applications ranging from altimetric and gravimetric corrections to data assimilation and model boundary forcings. The ArcTiCA dataset will serve as a springboard for the ocean tidal community to assess our current state-of-the-art models and advance future Arctic modelling efforts.

Finally, although we intend for this dataset to be a one-stop-shop for *in situ* tidal data in the Arctic Ocean, there are still significant gaps in the coverage of *in situ* measurements. Therefore, one aspiration of this manuscript is to motivate further concerted efforts to deploy more measurements and make existing measurements public for inclusion in updates to our dataset. As more time series are obtained, we will release updated versions of ArcTiCA aimed at increasing coverage and usability. These datasets will be documented within the README distributed with each new dataset version to allow users to find the data sources used. One additional dataset we intend to include in future iterations is the Alaska Department of Geological & Geophysical Surveys (DGGS) dataset (individual site data from https://water-level-watch.portal.aoos.org/); based on preliminary studies, this will require improved preprocessing techniques before tidal analysis and subsequent ingestion into ArcTiCA.

## Methods

### Data acquisition

The starting point for creating ArcTiCA was to acquire all available tidal height data, including sea level and bottom pressure time series, published tidal coefficients, and coefficients obtained by other researchers but unpublished.

As data coverage is scarce, we chose to include all data sources regardless of quality. Where possible, time-series data were preferred as they allowed us to apply a consistent set of quality controls and analysis techniques to estimate tidal constituents. Quality control included the removal of outliers and, where necessary, converting time series from local time to standardised UTC. Where we could not locate or obtain the original time series, we extracted constituents from existing databases and literature sources to expand the overall coverage of observations. Unlike the time-series data we analysed, these can only be quality controlled directly if the respective sources provided sufficient metadata, and only post-processing steps can be applied to these data.

**Fig. 1:**
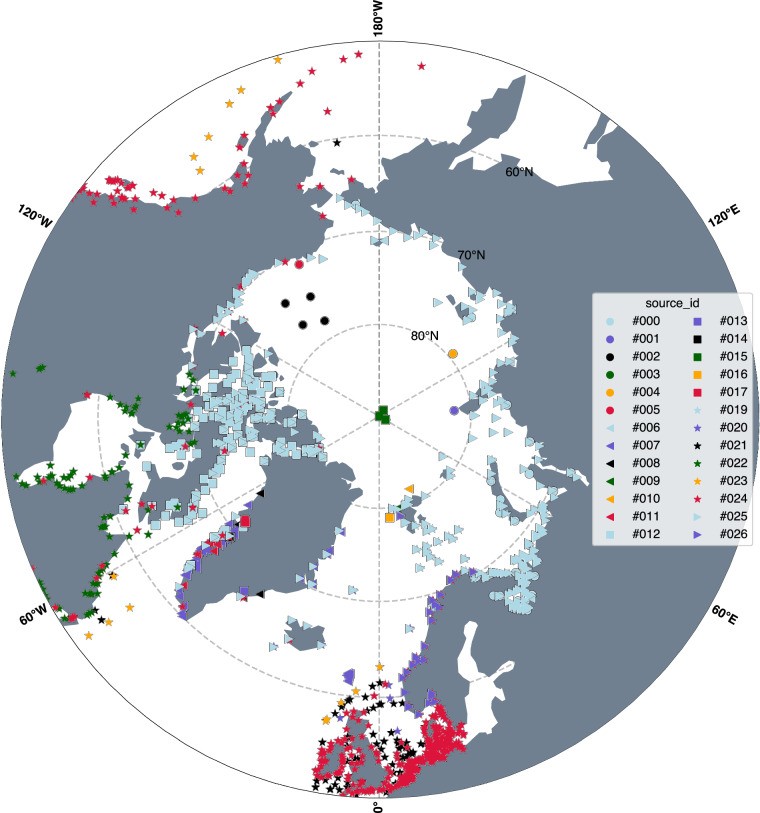
Distribution of data from each data source, with the Source IDs listed in Table 1.

A total of twenty-nine different data sources were identified that provide data in the Arctic Ocean from these three measurement types (Table 1), resulting in a total of 1624 stations (Fig. 1), with 914 of the stations being above 60°N. These data sources are provided in different manners: derived by us from time-series data, provided by previously published datasets, or taken from publications. The latter sources are not ideal for quality control purposes, but we determined that they should be included in ArcTiCA based on the scarcity of data in the region. Three measurement types are included in ArcTiCA and summarised in this manuscript: tide gauges, OBP sensors, and GNSS reflectometry.

**Table 1:** List of sources used in the creation of ArcTiCA as well as the type of data provided.

Source ID	Source	Source Data Type	Citation
#009	Kowalik_and_Proshutinsky_1994	Constituents	^2^
#010	Kulikov_etal_2018	Constituents	^47^
#016	Norwegian_Hydrographic_Service	Constituents and Time Series	https://www.kartverket.no/
#023	Stammer_etal_2014	Constituents	^8^
#025	Talibi_etal_2020	GNSS-IR Time Series	^39^
#001	Davis_etal_2014	Literature	^48^
#002	Dietrich_etal_2007	Literature	^49^
#004	Gjevik_and_Straume_1989	Literature	^50^
#005	Gjevik_etal_1992	Literature	^51^
#006	Greisman_etal_1986	Literature	^52^
#017	Peralta-Ferriz_etal_2014	Literature	^53^
#018	Peralta-Ferriz_2012	Literature	^54^
#021	Richter_etal_2011	Literature	^55^
#026	Voinov_2006	Literature	^56^
#028	Russian_Geographical_Society	Literature	https://elib.rgo.ru/
#003	Emily_Shroyer_pers_comms	OBP Time Series	Emily Shroyer, pers. comms.
#007	John_Mortensen_pers_comms	OBP Time Series	John Mortensen, pers. comms.
#008	Janout_etal_2023	OBP Time Series	^57^
#012	McRaven_and_Pickart_2022	OBP Time Series	^58^
#013	Morison_etal_2007	OBP Time Series	^59^
#014	Nilsen_etal_2021	OBP Time Series	^60^
#015	Frank_Nilsen_pers_comms	OBP Time Series	Frank Nilsen, pers. comms.
#019	Polyakov_2016	OBP Time Series	^61^
#020	Ray_2013	OBP Time Series	^26^
#022	Soren_Rysgaard_pers_comms	OBP Time Series	Soren Rysgaard, pers. comms.
#027	WHOI	OBP Time Series	^44^
#000	DMI	Tide Gauge Time Series	https://ocean.dmi.dk/tides^41–43^
#011	MEDS	Tide Gauge Time Series	https://isdm-gdsi.gc.ca/
#024	TICON-3	Tide Gauge Time Series	^30^

Tide gauges measure sea-level changes relative to a vertical datum, which usually varies between gauges. As their name suggests, they are used to study ocean tides, with the fixed position of these measurements and the frequent temporal sampling, usually hourly or shorter, allowing for the derivation of tidal constituents. Some older records were based on measurements of sea surface height at the times of high and low tides rather than the ocean height at regular time intervals; however, these data are not incorporated within this dataset. Tide gauges can be susceptible to non-ocean-related influences such as human interference, including instrument repositioning, vertical land motion, and instrument outages. Thousands of tide gauges have been deployed globally, although not particularly well distributed, by various institutions and governments, with several organised efforts being conducted to collect the variety of datasets based on varying applications, for example, GESLA-3^29^ and PSMSL^34^. Although these observations are of significant value, they are dominated by measurements at the coast, with only a few island stations globally providing insights into deep ocean tides. In the Arctic, there are no deep ocean island stations, and tides can vary substantially over the broad continental shelves of the Eurasian Arctic; therefore other data types are crucial to understanding Arctic tides.

Measurements of ocean tides by OBP sensors are a crucial part of this new tidal database. As OBP data require efforts to recover the sensor or to maintain an anchored mooring array, they are much less common than tide gauge observations, and their time series are typically shorter. OBP sensors are deployed mainly in the open ocean and shelf sea regions, making them crucial for filling regions with no tide gauge observations. There have been some previous efforts to compile tidal data from OBP measurements, including early work done under the auspices of the International Association for the Physical Sciences of the Ocean (IAPSO)^35–37^.

The Global Undersea Pressure (GLOUP) database, now housed within the U.K. National Tidal and Sea Level Facility, has also compiled many time series. However, it was last updated in the early 2000s. A compilation of high-quality tidal constants from OBP measurements by Ray^26^ has been used in several model validation studies^8,27^. However, that compilation is now ten years old, and several additional data sources have since been deployed or made publicly available in the Arctic Ocean.

Another *in situ* source becoming increasingly important over recent years is GNSS interferometric reflectometry (GNSS-IR). Although not the primary intention of GNSS-IR, ocean tidal constituents have been successfully estimated from sea-level measurements from GNSS-IR stations^38^. A study by Tabibi *et al*.^39^ presented tidal constituents from GNSS-IR measurements in both Greenland and Antarctica. These two regions have limited coverage from other *in situ* measurements; therefore, including GNSS measurements here is valuable.

### Data pre-processing

The methodology used in estimating tidal constituents depends on the data source. When the raw sea level or OBP data are available (for Source Data types containing ‘Time Series’), we use a common approach to the estimation of the tidal constituents. The publicly available software package *UTide*^40^ was used for consistency and reliability of the results and to allow for repeatability and well-defined processes for future updating of the dataset. Experiments using our own software (not shown), such as those used in creating TICON-3^30^, indicate that these packages provide essentially the same results for all the constituents of interest.

For the raw time-series data, outliers were removed if they deviated more than three times the standard deviation from the mean; this reduces the possibility that erroneous data will corrupt the tidal coefficient estimates. All the time series provided in local time were converted to UTC. This step is vital in preventing erroneous phase lag estimations within the dataset. The corrected time series were then used to produce suitable sets of tidal constituents based on the time-series length. As this length varies from gauge to gauge, with some being on the order of a few weeks or months and some being for multiple decades, only a certain number of tidal constituents can be estimated for all gauges. For tidal modelling efforts, the eight major tidal constituents (M_2_, S_2_, N_2_, K_2_, K_1_, O_1_, P_1_ and Q_1_) are of most interest in the Arctic and, therefore, when we estimate tides within this database from *in situ* time series, these constituents are always provided. The nodal modulation has been accounted for when deriving the constituents within this dataset using *UTide*. Note that the raw time series were not corrected for the inverse barometer effect.

For each *in situ* time series analysed by the authors, additional uncertainty information is provided based on the confidence interval information of the amplitudes and phases of each constituent, which is directly output by the *UTide* software. The confidence interval information is based on the coloured Monte Carlo method, which is the default selection within *UTide* and is further explained in^40^. The uncertainty or variance information provided by other sources is also included where appropriate, with the type of information being explained with an additional variable. The uncertainty information is provided to aid in the interpretation of the data provided within the dataset. For tide gauges and GNSS-IR instruments, the units for amplitude estimations are in centimetres (cm), while the OBP-derived amplitudes are provided in millibar (mbar).

The Danish Meteorological Institute (DMI) data^41–43^ are obtained from tidal height time series, which are themselves derived from estimated tidal constituents. These constituents are not publicly available, but DMI publishes tidal heights for two years from which tidal constituents can be estimated. Note that the time-series lengths to make the original tidal height time series are not publicly available. All other time series were taken as–is from the source, with the only exception being data from the WHOI source. These data were collected and made available by the Beaufort Gyre Exploration Program based at the Woods Hole Oceanographic Institution (https://www2.whoi.edu/site/beaufortgyre/) in collaboration with researchers from Fisheries and Oceans Canada at the Institute of Ocean Sciences^44^. These data have been collected in separate sampling efforts, usually deployed and recovered in one-year periods. To provide more reliable estimations of the tidal constituents, we combined data from these various deployments to create extended time series for the respective moorings.

### Data post-processing

**Fig. 2:**
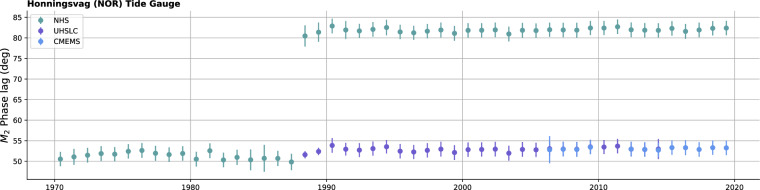
Yearly M_2_ phase estimations from three different GESLA-3 data sources of Honningsvag tide gauge.

Before producing the final dataset, an additional quality control step was performed on the time-series data from source types OBP Time Series, Tide Gauge Time Series and GNSS-IR Time Series. This was done by dividing the time series into yearly blocks and estimating constituents for each of these yearly blocks to check consistency, especially of phase lags. An example for a Norwegian tide gauge, Honningsvag, is provided in Fig. 2, illustrating the value of this procedure. For this location, the GESLA-3^29^ compilation includes data from three sources: the Norwegian Hydrographic Service (NHS), the University of Hawaii Sea Level Center (UHSLC), and Copernicus Marine Service (CMEMS). It is evident from Fig. 2 that there is a phase shift that occurs within the NHS estimations in 1988, and we determined that this was caused by a change in reference time from UTC pre-1988 to local time post-1988. The NHS data within GESLA-3 has this issue for several gauges (not shown). The cause of this issue is not fully diagnosed, but it occurred somehow during the construction of GESLA-3. At the time of publication, these Norwegian gauges in GESLA-3 have not been updated. Note that the NHS website also provides directly estimated constituents based on internally consistent tide gauge time series, i.e., based on one single time reference. Through a series of tests where we corrected individual gauges and compared them to constituent estimates taken directly from NHS, the use of the NHS-provided constituents was considered safe.

In some cases, tidal estimations were provided on time series in local times, which then required conversion to the UTC reference used throughout ArcTiCA. The standard formulation^45^ was used for the conversion based on the difference in hours between the local time zone and UTC and the frequency of the respective tidal constituent. Where identified, this conversion was done for each station and constituent.

The *in situ* constituents were also compared with global tide models to help further identify potentially incorrect phase lag estimations. Although models have uncertainty, the phase lag estimations can provide an adequate overall picture of the individual tidal constituents. This is illustrated in Fig. 4B,where the modelled DTU16 phase lag usually demonstrates overall consistency, within estimated error ranges, with *in situ* measurements. However, there are some evident exceptions. This step was particularly valuable for data sources that provided the constituents themselves, where we could not confirm the time references from the metadata. This step helped identify that the MEDS (#011) dataset used mainly across North America was all referenced to local time and, therefore, needed to be adjusted to be consistent with UTC throughout the dataset.

### Flag determination

**Fig. 3:**
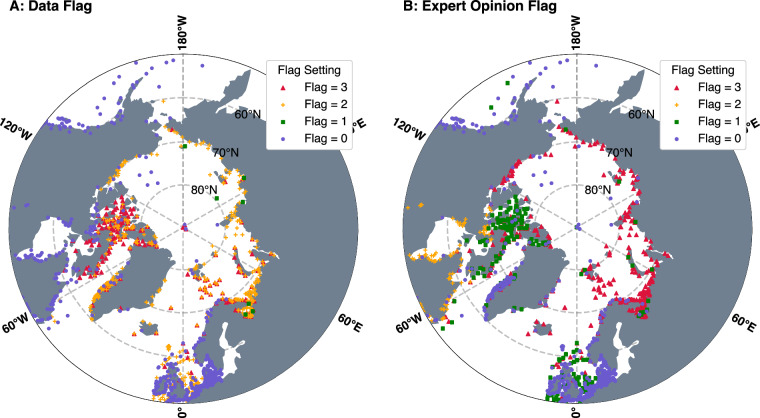
An illustration of both the (**A**) data and (**B**) expert opinion flag for the entire dataset, described in Table 2.

ArcTiCA provides two flags: a ‘data’ and an ‘expert opinion’ flag. The idea behind these flags is to allow an ArcTiCA user to determine the usefulness of each data record for their own applications. As several sources provide data from the same sites, these flags also help users decide which source of measurement they prefer. In the cases of both flags, the lower the given flag, the higher the confidence in the usefulness of the data. As tide data points in the Arctic are scarce, these flags should serve as a guideline for the entire dataset. A map of these two flags is given in Fig. 3, and a summary of the flags and the corresponding values are given in Table 2.

**Table 2:** Overview of the flags provided in ArcTiCA and appropriate descriptions of flags.

Flag Name	Flag No.	Description
*Data Flag*	0	Constituents derived from a time series.
	1	Constituents obtained directly from a source.
	2	Constituents taken from published datasets or websites.
	3	Constituents obtained from tables and figures in journal publications or reports.
*EO Flag*	0	*Excellent*; data and metadata is suitable for tidal estimations. Time-series length exceeds 1 year.
	1	*Good*; time-series length is shorter than 1 year but exceeds thirty days. Metadata indicates reliable estimations.
	2	*Fair*; insufficient meta data for a conclusion to be drawn on the appropriateness for tidal estimations.
	3	*Use with caution*; no meta data provided and there is concern on the accuracy of the provided information.

#### Data flag

The data source for the constituents is used to create the data flag. As the sources of data and techniques vary, this flag is designed as a general guide to a user who may place higher confidence in a particular source type. Where time-series data of either bottom pressure or sea level data are available from a gauge from which we could derive the estimations using consistent approaches, the data flag is 0.

Time series analysis by us is the preferred technique for deriving constituents within this dataset, as it also allows for the removal of outliers and accurate correction of the reference time of the measurement to UTC. A data flag value of 1 is given when the tidal constituents were obtained from a source directly. However, there is no way for us to check the accuracy of these tidal estimations; i.e., the time-series data was not directly available to us. A data flag of 2 is given when tidal coefficients were provided by external sources, meaning they were either taken from websites or published datasets, but where we cannot confirm the methodology of the estimations. These data are helpful, but certain provided measurements could still be imperfect. Finally, a data flag of 3 is given when constituents were extracted from published tables or figures from scientific literature. Again, these data cannot be directly assessed by us, and errors may be present within these data. Note that the data flag is not, by itself, a guarantee that the actual data quality of a specific record is better or worse than another with a different data flag. Several factors such as instrument drift and changing amplitudes and phases as sea ice varies, can influence the quality of individual measurements, regardless of whether the analyses were carried out by us (data flag = 0) or by someone else (data flags 1â€”3).

#### Expert opinion flag

The second flag is termed an ‘expert opinion’ flag (EO flag). It provides our assessment of the suitability of the different estimations based on available data or, for data flags 1–3, the detail in the metadata information. As with the data flag, this flag is not an indictment on the data itself; e.g., in some cases the metadata is insufficient to judge data quality, but the estimations themselves may still be accurate. It is the dataset user’s responsibility to review the original documentation and follow up with original data authors as needed. We encourage feedback to us on further insights into specific dataset elements that may change the EO flag for future ArcTiCA releases.

The EO flag is set to 0 when the data time series is analysed by us, is greater than one year long and the series has less than 10% missing data, allowing for reliable estimation and separation of major tidal constituents. An EO flag of 1 is given to measurements with less than one year’s worth of data but more than thirty days. This EO flag value is also given to data containing limited metadata but provided by sources with data flag = 1. This is done to account for estimations by previous studies where those authors did not provide the appropriate metadata required to determine this flag but are provided by sources that we trust based on their experience with tidal analyses. An EO flag of 2 is given to data where insufficient metadata is available, and, therefore, the reliability of the provided estimations cannot be determined. An EO flag of 3 is given when no metadata is provided, and there is a concern about the accuracy of the provided information, either related to the positional accuracy of the gauges or the tidal coefficients themselves. Additionally, record lengths that are less than thirty days are flagged as 3 as constituents determined from short records can contain large errors, particularly relating to *K*_1_ and *S*_2_.

## ArcTiCA Data Records

The ArcTiCA data are available from the Arctic Data Center at 10.18739/A2VT1GR64^46^ in either NETCDF4 or CSV formats. These formats are chosen to allow for ease of use by users and the selection of data by specific criteria. The crucial metadata users need to determine whether the tidal data is appropriate for specific applications, defined in Table 3, are provided within the dataset. Additionally, a README file (*ArcTiCA_README.pdf*) is provided and a history file (*ArcTiCA_Revision_History.txt*) is given for users to keep track of any updates that are released. Updates to ArcTiCA will be released with a new DOI. When new versions are released, the previous DOI will direct users to the newest version of ArcTiCA.

**Table 3:** The variables of the ArcTiCA dataset and a brief description of the variables.

Dataset variable	Description of variable
*source_id*	the provided ID number for the respective source
*lon*	the longitudinal position (in degrees 0 to 360) of the measurement
*lat*	the latitudinal position (in degrees) of the measurement
*cons*	the respective tidal constituent
*amp*	amplitude of the tidal constituent
*pha*	phase lag of the tidal constituent (from 0 to 360 degrees)
*amp_uncert*	the standard deviation of the amplitude (cm)
*pha_uncert*	the standard deviation of the phase lag (in degrees)
*start*	start time of the measurements
*end*	end time of the measurements
*number_of_obs*	the number of observations available within the *in situ* time series
*missing_obs*	the number of missing observations within a time series, i.e. gaps in the time series
*source*	the source of the tidal constants or time series used for tidal constituents estimation
*instrument*	the type of instrument: tide gauge, OBP or GNSS-IR
*site*	the site name of the instrument
*rec_length*	the total record length in days
*sampling_rate*	the sampling rate of the measurement in minutes
*inference*	whether inference methods are used in this measurement
*data_flag*	data flag as described in section *Data Flag*
*expert_flag*	expert opinion flag as described in section *Expert Opinion Flag*
*site_record*	the observations record number for the particular site
*site_total*	the total number of sources for the same particular site
*amp_units*	the units of the amplitude estimations (cm or mbar)
*uncert_info*	the type of uncertainty information provided, either standard deviations (STD), confidence intervals (CI) or none.
*notes*	any notes about this particular station

**Fig. 4:**
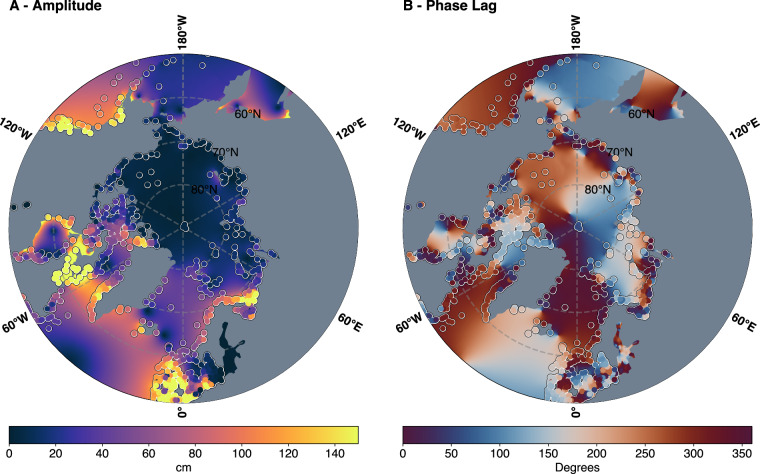
The distribution of tidal constants from the ArcTiCA dataset overlaid onto an M_2_ tidal amplitude and phase estimate from DTU16.

At the time of initial publication, ArcTiCA contains 1924 individual *in situ* stations with at least an estimation for the M_2_ constituent and 1020 with at least eight constituents. A total of 29 data sources were used to create the current dataset, where either the tidal constituents were directly estimated by us, or the data were provided from personal communications or literature sources. Where possible, all available metadata from the different sources is provided within the dataset, which is shown in Table 3. The current spatial distribution of 1924 *in situ* stations is presented in Fig. 4 for the amplitude and phase lag of the M_2_ tidal constituent and compared to a global tide model, DTU16^28^.

Although duplicates remain due to obtaining data for the same site from different sources, they are kept within ArcTiCA as they often contain estimations from varying sources or use different time-series lengths. It is the user’s responsibility to decide which source to use for individual sites using the recommendations provided by the data and EO flags. For each site, all the available constituents are given; therefore, sites have differing numbers of constituents. A total of 43 different tidal constituents are available within ArcTiCA. This decision was preferred over limiting the dataset to a certain number of constituents to provide as much data as possible for future modelling efforts, which will continue to expand the number of tidal constituents investigated.

## Technical Validation

### Evaluation using a model ensemble

**Fig. 5:**
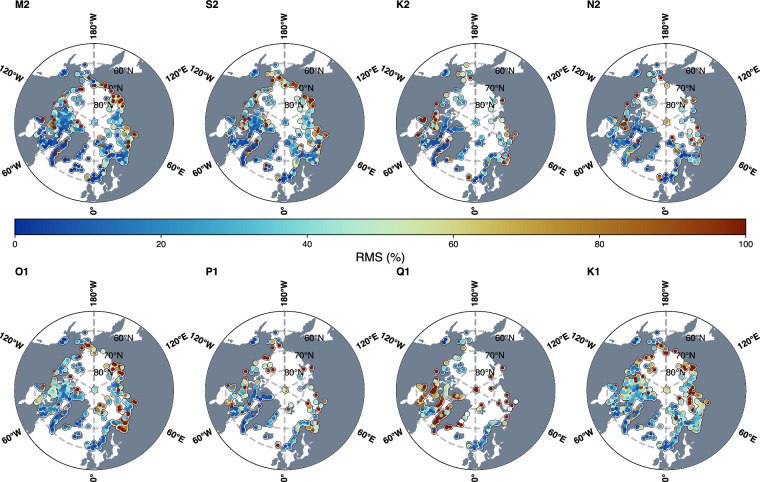
RMS of an ensemble of models (DTU16, GOT5.1, EOT-Polar v0.1 and FES2014b) relative to the respective amplitudes for the eight major tidal constituents taken from the ArcTiCA dataset.

We used the M_2_ coefficients from all available *in situ* measurements for our initial evaluation of ArcTiCA. Figure 5 demonstrates the overall differences between an ensemble of models for the M_2_ constituent in terms of root-mean-square error (RMS), as well as amplitude and phase differences. This comparison demonstrates the value of these flags, as regions with extreme differences between observations in the models are likely those that would be flagged within the dataset.

The ensemble of ocean tide models was estimated by taking the mean amplitude and phase calculated from four tide models; DTU16^28^, GOT5.1 (^26^ updated), EOT-Polar-v0.1 (^27^ updated) and FES2014b^17^. The models differ in terms of the data sets that are assimilated; for records in ArcTiCA that have been assimilated in a particular model, we expect reduced errors compared with models with no assimilation of that site. We chose an ensemble approach to account for individual model errors that could influence statistical interpretations and to avoid an inter-model comparison that is beyond the scope of this dataset documentation. Note that, even for models in the lower latitudes with abundant observations and satellite data, the coastal region remains the most challenging region for ocean tide models. Since most observations are tide gauges at the coast, we expect that model and observations would have differences that exceed centimetres^8,27^ This is due to factors such as limited bathymetry information, complex tidal dynamics and sea-ice interactions. Additionally, many gauges are in river or estuarine environments, including fjords, where the relatively coarse grids of the global models do not provide ocean tide estimates.

The model ensemble’s median RMS for M_2_ was 5.89 cm for the dataset above 60°N and 4.63 cm above 70°N, which correlates well with previous literature. Cancet *et al*.^9^ and Stammer *et al*.^8^ both independently evaluated global tide models against *in situ* stations in the Arctic, although using only 121 and 20 tide gauge measurements, respectively. Stammer *et al*.^8^ reported RMS errors for the M_2_ tide between 3.91 and 5.89 cm, while Cancet *et al*.^9^ found errors between 5.8 and 8.7 cm, both using some of the major global ocean tide models. For the other constituents, these RMS differences align well with previous literature. The regions with the highest RMS were along the Russian coastline and within Hudson Bay. The latter is a well-known region of difficulty for tide models, having a relatively high spread between tidal models in this region^8^, and the RMS in this region is primarily driven by differences in amplitudes between the measurements and the models.

On the other hand, the Russian coastline is a complex tidal region and has troubled modellers due to limited bathymetry information. However, the RMS difference here may additionally be explained by the relatively poor quality of the situ measurements, which are either extremely old or are provided with very little metadata to confirm their estimations. When making use of the EO flag by selecting only data flagged as 0 and 1, the RMS decreases to 5.20 and 4.31 cm above 60°N and above 70°N, respectively, which remains in line with previous literature. This is primarily due to the removal of several gauges with an EO flag of 2 or 3 (see Fig. 3), which further highlights the importance of both the data and EO flag in the application and interpretation of the dataset.

## Usage Notes

ArcTiCA will serve the ocean tide modelling community by providing extensive model validation data and by offering a substantially larger database for assimilation into inverse models. Although we have attempted to make comprehensive flags and remove unreliable or erroneous estimations, users should still proceed with caution when drawing their own conclusions from using these data. In these cases, we strongly recommend that users consider the *data_flags* and *expert_flags* within ArcTiCA when interpreting their results for a specific region. In some cases, users may find that high-quality data are very sparse and that a model that assimilates tidal coefficients obtained from satellite altimetry may provide higher-accuracy coefficients than can be obtained from a poorly-located and essentially undocumented tide gauge record.

ArcTiCA is designed to make using the data as easy to do as possible by using formats that are easily ingested by any popular coding software. Examples of how to use the dataset and select data by specific variable criteria are provided in a public GitHub repository found at https://github.com/hart-davis/ARCTiCA/.

## Data Availability

A public GitHub repository has been set up to help users process the data, which will be updated based on user feedback or input as well as when changes occur to the dataset. This repository can be found at https://github.com/hart-davis/ArcTiCA.
